# Safety and Feasibility of Transcaval Access for the Delivery of Impella Microaxial Flow Pump

**DOI:** 10.1016/j.jscai.2025.103789

**Published:** 2025-07-31

**Authors:** Mustafa Mohammed, Waleed Al-Darzi, Ahmad Jabri, Laith Alhuneafat, Ahmed Kazem, Pedro Engel Gonzalez, Tiberio Frisoli, Khaldoon Alaswad, Mir Babar Basir, Mohammad Alqarqaz, Brian O’Neill, James Lee, William W. O’Neill, Pedro Villablanca

**Affiliations:** a Division of Cardiology, Henry Ford Health, Detroit, Michigan; b Division of Cardiology, University of Minnesota, Minneapolis, Minnesota; c Center for Structural Heart Disease, Henry Ford Health, Detroit, Michigan

**Keywords:** cardiogenic shock, Impella, mechanical circulatory support, transcaval access

## Abstract

**Background:**

Transcaval access (TCA) may enable percutaneous mechanical circulatory support (MCS) with reduced risk of vascular complications in cardiogenic shock patients needing mechanical support.

**Methods:**

This single-center retrospective study included patients who underwent TCA placement of an Impella 5.0 (Abiomed) from June 2015 to March 2023. Data on demographic characteristics, clinical, procedural variables, and in-hospital outcomes were collected.

**Results:**

Seventy-two patients (mean age, 58.2 years; 66.7% men) were included. Twenty-eight patients had nonischemic cardiomyopathy and 43 had ischemic cardiomyopathy, with a baseline left ventricular ejection fraction of 23.5% ± 14.2%. Most patients (90.3%) were in categories C to E of the Society for Cardiovascular Angiography & Interventions (SCAI) classification for cardiogenic shock. TCA and MCS delivery were successful in all cases. Forty-two patients survived to explant device and TCA sheath, with successful explant in 36 using nitinol occluders; 7 needed a covered stent due to underlying right ventricular dysfunction to avoid right ventricular failure. Overall, in-hospital survival was 44.4%, with 43.8% in the nonischemic group and 56.2% in the ischemic group. Bleeding Academic Research Consortium (BARC) bleeding >1 occurred in 13.9%. No vascular complications from the access site were observed. During hospitalization, 16.7% had ventricular tachycardia/ventricular fibrillation and 5.6% had pulseless electrical activity postimplantation. Acute kidney injury requiring hemodialysis occurred in 15.3%, and 4.2% had a stroke. The average length of stay was 19.9 days (IQR, 3-28.25).

**Conclusions:**

Transcaval access for Impella 5.0 is safe and feasible in experienced hands for patients needing advanced MCS due to inadequate peripheral arterial access or insufficient support from conventional devices.

## Introduction

Cardiogenic shock is a clinical condition defined by an insult to the cardiac function, causing a reduction in myocardial contractility, hypotension, diminished cardiac output, and resultant organ ischemia.^1^ Despite our advancing therapies utilized in the setting of cardiogenic shock, associated mortality is as high as 70% in certain settings.^2^ When noninvasive treatments such as vasopressors and inotropic medications fail, mechanical circulatory support (MCS) is used to provide hemodynamic support. These invasive therapies include the intraaortic balloon pump, Impella (Abiomed), TandemHeart (LivaNova), as well as veno-arterial extracorporeal membrane oxygenation (ECMO).^3^^,^^4^ Compared to intraaortic balloon pumps, Impella devices offer significant cardiac output augmentation, with the Impella 2.5 and CP providing 2.5 to 3.5 L/min.^5^ In refractory shock, however, additional left ventricular offloading may be needed. In cases of refractory shock, the Impella 5.0, capable of delivering up to 5 L/min, offers enhanced left ventricular support and improved hemodynamic parameters.^6^

Traditionally, Impella 5.0 devices are implanted surgically via the axillary or femoral arteries. However, surgical placement in critically ill patients poses significant challenges, and arterial access complications are associated with substantial morbidity and mortality.^7^ As an alternative, transcaval (caval-aortic) access (TCA) has emerged, allowing venous entry through the femoral vein and inferior vena cava into the abdominal aorta. This method facilitates transcatheter procedures and mechanical support device placement in patients with poor arterial access due to severe peripheral arterial disease.^8^^,^^9^ Previous studies have demonstrated the safety and feasibility of TCA for transcatheter aortic valve replacement (TAVR) in patients with inadequate arterial access.^10^ A recent case series suggested TCA for MCS in cardiogenic shock as a viable option.^9^

This study aims to evaluate the safety, feasibility, and outcomes of using TCA for Impella 5.0 MCS in patients with ischemic and nonischemic systolic cardiogenic shock, providing further insights into this novel approach.

## Materials and methods

### Study design and patient selection

This is a single-center retrospective study conducted at a large academic institution. Data were retrospectively collected from June 2015 to March 2023. The study aimed to evaluate the safety, feasibility, and outcomes of TCA for Impella 5.0 in patients with ischemic and nonischemic systolic acute heart failure.

Patients included in the study were those who required MCS due to cardiogenic shock and had the Impella 5.0 device placed via TCA. Cardiogenic shock was defined according to the Society for Cardiovascular Angiography & Interventions (SCAI) shock classification schema categories C-E. The study did not select patients based on the underlying cause of cardiogenic shock, allowing for a diverse patient population. Institutional practice for TCA selection is based on predefined criteria, including severe peripheral arterial disease, small caliber arteries (<6 mm), prior limb ischemia from MCS, or inadequate hemodynamic support with conventional devices requiring upgrade to 5.0 Impella. These factors are assessed via clinical evaluation, ultrasound, angiogram, and/or prior imaging when available (Table 1, Central Illustration).

**Table 1:** Patient characteristics.

	Total (N = 72)	Ischemic cardiomyopathy (n = 43)	Nonischemic cardiomyopathy (n = 29)
Age, y	58.2 ± 12.7	62.9 ± 10.5	51.1 ± 12.6
Male sex	48 (66.7%)	32 (74.4%)	16 (55.2%)
Race			
White	51 (70.8%)	35 (81.4%)	16 (55.2%)
African American	12 (16.7%)	3 (7%)	9 (31%)
Other/unknown	9 (12.5%)	5 (11.6%)	4 (13.8%)
Comorbidities			
Hypertension	47 (66.2%)^a^	29 (69%)^a^	18 (62.1%)
Diabetes mellitus	30 (42.3%)^a^	21 (50%)^a^	9 (31%)
Hyperlipidemia	43 (60.6%)^a^	27 (64.3)^a^	16 (55.2%)
Tobacco smoking	31 (43.7%)^a^	21 (50%)^a^	10 (34.5%)
Obstructive sleep apnea	11 (15.5%)^a^	4 (9.5%)^a^	7 (24.1%)
Chronic obstructive lung disease	10 (14.1%)^a^	8 (19%)^a^	2 (6.9%)
Previous myocardial infarction	29 (40.8%)^a^	26 (61.9%)^a^	3 (10.3%)
Previous cerebrovascular accident	8 (11.3%)^a^	5 (11.9%)^a^	3 (10.3%)
Atrial fibrillation	21 (29.6%)^a^	8 (19%)^a^	13 (44.8%)
HFrEF	38 (53.5%)^a^	21 (50%)	17 (58.6%)
Left ventricular ejection fraction, %	29 ± 17.8^b^	28.4 ± 16.3	30.1 ± 19.8
Right ventricular dysfunction	15 (31.3%)^c^	8 (30.8%)	7 (31.8%)
Creatinine prior to Impella implant, mg/dL	2.5 ±1.5^d^	2.24 ± 1.37	2.81 ± 1.7
Lactate prior to Impella implant, mmol/L	5.5 ± 4.8^e^	5.9 ± 4.7	4.9 ± 5
Inotropes prior to Impella	36 (50%)	17 (39.5%)	19 (65.5%)
Pressors prior to Impella implant	41 (57%)	23 (53.5%)	18 (62.1%)
Prior mechanical support device (n = 36)			
IABP	17 (23.6%)	12 (27.9%)	5 (17.2%)
Impella 2.5/CP	17 (23.6%)	11 (25.6%)	6 (20.7%)
Impella 5.0 (arterial access)	2 (2.8%)	2 (2.8%)	0
Reported reasons for using an alternative access			
Peripheral vascular disease	12 (16.7%)	11 (25.6%)	1 (3.5%)
Small caliber arteries	20 (27.8%)	10 (23.25%)	10 (34.5%)
Inadequate hemodynamic support	16 (22.2%)	10 (23.25%)	6 (20.7%)
High-risk PCI	5 (6.9%)	5 (11.6%)	0
Limb ischemia from prior MCS	5 (6.9%)	3 (7%)	2 (6.9%)
Unclear reason	11 (15.3%)	4 (9.3%)	7 (24.1%)
Other	3 (4.2%)	0	3 (10.3%)

Values are mean ± SD or n (%).

HFrEF, heart failure with reduced ejection fraction; IABP, intra-aortic balloon pump; MCS, mechanical circulatory support; PCI, percutaneous coronary intervention.

a One patient had an unknown medical history.

b Ejection fraction was unavailable for 28 total patients (19 in ischemic cardiomyopathy).

c Right ventricular dysfunction data were unavailable for 24 patients (17 in ischemic cardiomyopathy).

d Missing 3 data points all in the ischemic cardiomyopathy group.

e Missing 14 data points (11 in ischemic cardiomyopathy); analysis done on available data.

**Central Illustration:**
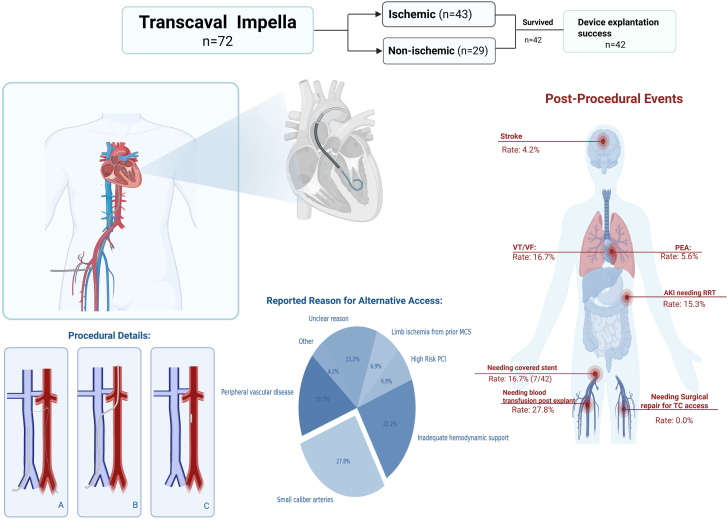
**Asummary of the transcaval (TC) Impella experience, including overall survival, a simple illustration of the procedure, reasons for the use of TC access, and postprocedural events.** AKI, acute kidney injury; MCS, mechanical circulatory support; PCI, percutaneous coronary intervention; PEA, pulseless electrical activity; RRT, renal replacement therapy; VF, ventricular fibrillation; VT, ventricular tachycardia.

### Data collection

Demographic characteristics, clinical, and procedural variables were collected, including age, sex, etiology of cardiomyopathy (ischemic or nonischemic), baseline left ventricular ejection fraction, SCAI shock classification, use of inotropes and pressors, and presence of prior MCS devices. In-hospital outcomes, such as survival to device explantation, complications, and length of stay, were also documented.

### TCA technique

The TCA procedure was performed by experienced interventional cardiologists. The technique for TCA has been described in previous studies.^8^^,^^9^^,^^11^^,^^12^ Supplemental Appendix S1 contain the detailed procedural steps and a simple illustration is provided in the Central Illustration. The following steps outline the general approach:

1. Preprocedural planning: Whenever feasible, preprocedural computed tomography (CT) imaging was obtained to identify suitable access sites. However, in many cases, the emergent need for MCS precluded the use of CT imaging.
2. Access site preparation: Femoral venous and arterial access was obtained under fluoroscopic guidance. The procedure began with the insertion of a guide wire into the femoral vein, which was advanced to the inferior vena cava.
3. Caval-aortic crossing: Using fluoroscopy and anatomical landmarks, a caval guide wire was electrified and advanced through the wall of the inferior vena cava into the adjacent abdominal aorta. This step was critical for creating a controlled connection between the venous and arterial systems.
4. Sheath insertion: After successfully crossing into the aorta, the guide wire was exchanged for a rigid guide wire, and a 22F or 24F sheath was introduced into the aorta. The Impella 5.0 device was then advanced through the sheath and positioned in the left ventricle.
5. Device placement: The Impella 5.0 was placed and activated to provide MCS, delivering up to 5 L/min of cardiac output augmentation.

### Closure technique

Upon completion of the support period, the Impella device and TCA sheath were removed. Closure of the TCA site was achieved using nitinol occluders in the majority of cases. In instances where residual fistulas posed a risk of right ventricular failure, covered stents were deployed at the arteriotomy site. The closure technique aimed to minimize bleeding and other complications.

### Ethical considerations

The study protocol (no. 14675) was approved in March 2021 by the Henry Ford Health System Institutional Review Board. Due to the retrospective nature of the study and the use of deidentified patient data, informed consent was not required. All procedures were performed in compliance with relevant institutional guidelines. The study adhered to the ethical standards of the 1964 Declaration of Helsinki and its later amendments, as well as the Health Insurance Portability and Accountability Act privacy requirements.

### Statistical analysis

Data were analyzed using SPSS (IBM Corp). Continuous variables were presented as mean ± SD, and categorical variables were presented as count and percentage. Procedural success, in-hospital outcomes, and complication rates were calculated for the entire cohort and subgroups based on the etiology of cardiomyopathy (ischemic vs nonischemic). Statistical comparisons were not performed due to the descriptive nature of the study and the relatively small sample size.

### Results reporting

Baseline characteristics, procedural details, and clinical outcomes are reported in tabular and narrative formats. Specific outcomes of interest included procedural success, survival to device explantation, complication rates, and length of hospital stay. Additionally, the impact of preprocedural CT imaging on procedural success and complication rates was evaluated.

## Results

### Patient demographics and baseline characteristics

A total of 72 patients who underwent TCA Impella 5.0 placement were included in the study. The cohort had an average age of 58.2 ± 12.7 years and comprised predominantly men (66.7%, n = 48) and White (70.8%, n = 51). Among these patients, 43 (59.7%) had ischemic cardiomyopathy, whereas 28 (38.9%) had nonischemic cardiomyopathy. The baseline left ventricular ejection fraction was significantly reduced, with an average of 23.5% ± 14.2%. Most patients (90.3%) were classified in categories C to E of the SCAI shock classification schema for cardiogenic shock. Additionally, 50% of the patients required inotropes, and 56.9% required pressors prior to the procedure. Prior to TCA, 51.4% of the patients had an MCS device already in place. Only 13.9% of the patients had preprocedural CT imaging reviewed for planning (Table 1). Available pre- and post-Impella hemodynamics are summarized in Table 2.

**Table 2:** Summary of hemodynamics prior to and post-Impella 5.0 placement via transcaval access.

Hemodynamic parameter	Prior to implantation	Postimplantation	*P* value
Right atrial pressure, mm Hg	15.75 ± 6.97	13.75 ± 6.49	.149^a^
Pulmonary arterial pressure, mm Hg	34.49 ± 10.1	28.98 ± 9.27	.002^a^
Pulmonary artery pulsatility index	1.9 ± 1.40	1.72 ± 1.19	.377^a^
Cardiac output, L/min	4.00 ± 1.58	5.06 ± 1.71	<.001^a^
Cardiac index, L/min/m^2^	2.00 ± 0.83	2.56 ± 0.84	<.001^a^
Systemic arterial pressure, mm Hg	72.35 ± 13.77	78.42 ± 12.48	.007^a^
Cardiac power output, W	0.617 ± 0.2617	0.861 ± 0.2789	<.001^a^
Cardiac power output deficit	0.58989 ± 1.353	–0.36615 ± 1.929	<.001^a^

Values are mean ± SD.

a Data available for analysis between 47-57 patients.

### Procedural details and outcomes

The TCA and MCS delivery were successful in all attempted cases. The average duration of Impella 5.0 support was 5.48 ± 6.29 days.

Of the total cohort, 42 patients (58.3%) survived to explant the device and TCA sheath. Explantation was successful in 36 patients using nitinol occluders, whereas 7 patients required a covered stent at the arteriotomy site to avoid right ventricular failure due to fistula flow in the setting of underlying right ventricular dysfunction. Specifically, 4 of these patients had no occluder initially, 1 was upgraded to tandem ECMO, and 1 was transitioned to comfort care and had the device removed without an occluder or stent. No surgical repair was necessary for any of the patients. All residual fistulous tracks were graded as ≤2 (Table 3).

**Table 3:** Procedural characteristics.

Characteristics	Counts (N = 72)
Impella explanted successfully	42
Device utilized for closure	
Amplatzer Duct Occluder 10/8 mm	22
Amplatzer Duct Occluder 10/12 mm	11
Amplatzer Muscular VSD 8 mm	1
Amplatzer Muscular VSD 9 mm	1
Amplatzer Muscular VSD 12 mm	1
Covered stent in aorta	7
Type of residual fistula tracks	
0	9
1	20
2	6
3	1
BARC bleeding type from Impella insertion-removal site	
0	48
1	14
2	3
>2	7
Size of the sheath used	
22F	55
24F	17

BARC, Bleeding Academic Research Consortium.

The in-hospital survival rate was 44.4% for the entire cohort, with a survival rate of 43.8% (n = 14) for the nonischemic group and 56.2% (n = 18) for the ischemic group. Significant bleeding (BARC >1) from the Impella insertion/removal site was observed in 13.9% of patients. There were a total of 6 retroperitoneal bleeding events but it was unclear if they were related to prior access or TCA. One was a large bleed, and the patient underwent an aortogram after Impella removal that noted contrast extravasation around TCA. Three other ones were self-limited, and only one of those required transfusion. The last one was small but also had a small 2.8 mm pseudoaneurysm in the abdominal aorta at the level of the pancreatic head that was eventually coiled without complications. Only one of those bleeds occurred in a patient who had preprocedural CT and the bleed was self-limited. During hospitalization, 16.7% of the patients experienced ventricular tachycardia or ventricular fibrillation, and 5.6% experienced pulseless electrical activity postimplantation. Acute kidney injury requiring hemodialysis occurred in 15.3% of patients, and 4.2% had a stroke (Table 4, Central Illustration). The average length of hospital stay for the entire cohort was 19.9 days (IQR, 3-28.25).

**Table 4:** Postprocedural complications.

Complications	Total (N = 72)	Ischemic cardiomyopathy (n = 43)	Nonischemic cardiomyopathy (n = 29)
Cerebrovascular accident	3 (4.2%)	1 (2.3%)	2 (6.9%)
Ventricular tachycardia/ventricular fibrillation	12 (16.7%)	8 (18.6%)	4 (13.8%)
Pulseless electrical activity	4 (5.6%)	4 (9.3%)	0
Acute renal injury after placement	23 (31.9%)	13 (30.2%)	10 (34.5%)
Acute renal injury after placement requiring renal replacement therapy	11 (15.3%)	3 (7%)	8 (27.6%)
Patients requiring blood transfusions postexplant	20 (27.8%)	12 (28%)	8 (27.6%)
Surgical repair required for the transcaval access	0	0	0

Of the 32 patients who survived, 21 had initially started with Impella 5.0 without any prior MCS. For those who died during follow-up, causes included COVID-19 pneumonia leading to undifferentiated shock, elective transition to home hospice with milrinone, deactivation of a left ventricular assist device due to poor quality of life, and unknown causes in 2 patients who died at home (Table 5).

**Table 5:** Clinical outcomes.

Outcome	Counts
In-hospital mortality	40/72
CHF admissions postdischarge	7/32
Mortality on follow-up (range 12-74 mo)	5/32
Outcomes of patients who survived to discharge (n = 32)	
Transplant	1
LVAD	2
LVAD then transplant	2
Recovery	11
PCI	10
PCI and valve intervention	2
Valve intervention	2
Home hospice on milrinone	1

CHF, congestive heart failure; LVAD, left ventricular assist device; PCI, percutaneous coronary intervention.

## Discussion

Transcaval access, first pioneered at the National Institutes of Health in 2010 to address delivery issues with large-bore devices, underwent refinement and animal testing before its first human TAVR was performed by Greenbaum et al^8^ in 2013 at Henry Ford Hospital.^13^ Our study presents the largest cohorts to date evaluating the use of TCA for the placement of the Impella 5.0 device in patients with cardiogenic shock. The findings indicate that TCA is a feasible and safe approach for MCS in a population with significant hemodynamic compromise and limited options for traditional arterial access. Despite the high baseline risk and complexity of the patient population, TCA was successfully performed in all cases, demonstrating its potential as an alternative to surgical or percutaneous arterial access. The in-hospital survival rates and complication profiles offer critical insights into the procedure's viability, particularly in settings where conventional methods are not feasible.

The procedure was successful in all attempted cases in our study, and the device was delivered effectively to the entire cohort. Similarly, a multicenter prospective registry of TCA for TAVR demonstrated a 99% success rate.^12^^,^^14^ Remarkably, despite a significant portion of patients not undergoing preplanning imaging given the emergent nature in most cases, transcaval procedures proved highly successful. Only 10 (13.9%) patients underwent preprocedure CT, and despite that, transcaval puncture between L3 to L5 proved safe. This underscores the viability of these procedures, especially in situations where time constraints, often due to the critical nature of cardiogenic shock, prevent preprocedural imaging, though CT remains preferred for elective cases when feasible.

Importantly, significant bleeding (defined as BARC >1) from the Impella insertion/removal site was reported in 13.9% of patients. In a review article assessing Impella-related complications, 12 studies (54.5%) reported major bleeding events without specifying the bleeding site.^15^ The low rate of access-related bleeding complications and the absence of vascular complications in our study support the safety profile of TCA. Notably, most patients had noncalcific aortas, which is likely one of the reasons for the low bleeding risk. According to the transcaval TAVR registry, there was a 7% incidence of life-threatening bleeding based on the Valve Academic Research Consortium-2 criteria.^12^ One challenge when comparing bleeding rates in studies involving large-bore devices is the variation in the definition of major bleeding across different studies. Data from the TAVR registry regarding TCA revealed a 13% incidence of major vascular complications during the procedure. There were no subsequent vascular complications between discharge and 30 days.^12^ However, our study did not evaluate long-term outcomes. More recently, the 1-year results of this population were published, including a comprehensive CT analysis, and remarkably, there were no late bleeding or vascular complication events related to the access point or closure device.^11^^,^^16^

Conventional TAVR and endovascular aneurysm repair procedures necessitate large-bore access, inherently heightening the risk of vascular complications and bleeding. Despite reductions in device size, these complications remain common, with reported rates as high as 20% for TAVR and 12% to 22% for endovascular aneurysm repair.^15^^,^^17^, ^18^, ^19^ When compared to traditional femoral arterial access and other MCS devices, such as ECMO and the Impella CP, TCA for Impella 5.0 shows promising results. Traditional femoral arterial access for MCS can be associated with higher rates of vascular complications, which were notably absent in this study’s TCA cohort. A review article examining Impella-related complications revealed a major vascular complication rate of 2.6% (range, 0%-8.3%), with 15 out of 19 studies reporting rates below 5%.^15^ This study encompassed a diverse patient group with different types and sizes of Impella devices. Another study involving 229 patients on veno-arterial ECMO showed nearly 34% vascular complications among those undergoing peripheral cannulation.^20^

Only 7 patients required covered stents given underlying right ventricle dysfunction in order to reduce the risk of right ventricular failure from residual shunt, indicating that the risk of significant complications can be managed with appropriate techniques. The use of nitinol occluders for closure was effective in most cases, reducing the need for more invasive measures. Caval-aortic fistulas are generally well tolerated and typically close within days to weeks as the nitinol occluder thromboses. Recent 1-year outcomes from the initial transcaval registry revealed no late occluder fractures or migrations, and only 1 persistent fistula in a patient with a misplaced aortic occlusion device.^11^

The ability of the Impella 5.0 to provide up to 5 L/min of cardiac output augmentation offers a significant advantage over smaller devices, making it particularly valuable in patients with severe hemodynamic compromise. This is especially relevant in refractory cardiogenic shock, where additional left ventricular offloading is critical. Our patients experienced high mortality rates as high as 56%, which is consistent with the literature reporting 40% to 60% mortality rates in cardiogenic shock patients.^2^^,^^21^^,^^22^ Moreover, patients with very poor vascular access requiring TCA may have more comorbidities than other patients with cardiogenic shock. Notably, our ischemic and nonischemic groups had comparable in-hospital mortality (56.2% vs 43.8%, *P* = NS), aligning with recent literature suggesting heterogeneous outcomes based on shock etiology.^23^ The small sample size precludes definitive conclusions, but the trend mirrors the DanGer Shock trial’s findings of Impella benefit in ischemic shock.^24^ This may warrant further investigation into patient selection criteria and the underlying pathophysiology of different cardiomyopathies.

The establishment of specialized shock centers could play a pivotal role in improving outcomes for patients with cardiogenic shock. These centers, equipped with the necessary expertise and advanced technologies for MCS, can facilitate early intervention and optimize patient management. Early transfer to such centers allows for the timely use of advanced support techniques like TCA for Impella 5.0 placement, potentially improving survival rates and reducing complications. The concept of shock centers is supported by studies indicating that early and specialized care is associated with better outcomes in severe cardiogenic shock.^25^ As for the adoption of TCA use, we propose it as the preferred approach for patients with poor arterial access but not universally for all cases until further multicenter data validate its safety in broader populations and only with available specialized expertise. We strongly recommend proctoring for initial cases including collaboration with experienced centers and utilizing existing expertise in the field.

Unfortunately, the Impella 5.0 has been discontinued from the market. Still, the utility of TCA in the use of MCS and delivery of TAVR remains. The technique can be employed with the use of ECMO and Impella CP. This is not applicable with Impella 5.5, given the short catheter length which is designed for axillary access. Our single-center experience has proved the safety and feasibility of TCA for MCS over the past several years including transcaval ECMO and others in prior publications.^26^, ^27^, ^28^, ^29^

### Limitations

There are several limitations to this study that need to be acknowledged. The retrospective design limits the ability to control for all potential confounding variables, as data were collected from electronic health records, which may be subject to inaccuracies or incomplete documentation. Additionally, the study was conducted at a single academic institution that more routinely performs transcaval TAVR, which means that the findings may not be generalizable to other settings. The expertise and procedural success observed at this center may not reflect broader clinical practice. Therefore, centers new to TCA should use prior established protocols and guidelines along with collaboration with experienced centers.^12^ Patient selection bias is another important consideration. Patients were included based on the availability of data and clinical indications for TCA, which may introduce selection bias. The decision to use TCA over traditional methods was likely influenced by patient-specific factors that are not fully accounted for. Moreover, the relatively new nature of the TCA technique implies a learning curve for operators. Initial procedures might have had different outcomes compared to those performed later in the study period as proficiency increased. Importantly, the study population was very sick and included many who needed an upgrade of mechanical support highlighting the critical nature.

Furthermore, the study primarily focuses on in-hospital outcomes and does not provide detailed information on long-term survival and quality of life postdischarge. Long-term follow-up is necessary to fully understand the implications of TCA for Impella 5.0 placement. Lastly, the cohort included patients with various types of cardiomyopathies and different stages of cardiogenic shock. This heterogeneity makes it challenging to draw definitive conclusions about the effectiveness of TCA in specific subgroups.

## Conclusion

This study demonstrates that TCA for Impella 5.0 placement is a viable and safe alternative for providing MCS in patients with cardiogenic shock, particularly those with limited peripheral arterial access. The procedure shows a high success rate and manageable complication profile, underscoring its potential in advanced cardiac care. Future research should focus on multicenter studies, long-term outcomes, and the development of standardized protocols to further validate and optimize this approach.
