# Supplementary material for: Safety and Feasibility of Transcaval Access for the Delivery of Impella Microaxial Flow Pump
Source: J Soc Cardiovasc Angiogr Interv. 2025 Jul 31;4(10Part A):103789. doi: 10.1016/j.jscai.2025.103789 (PMC12629743; doi:10.1016/j.jscai.2025.103789)
Supplement: Supplemental Appendix S1 [file mmc1.docx]

Procedural Details:

Key technical points:

Based on the methodology described in the PMC article *[Greenbaum AB et al. (2016) "Caval-aortic access to allow transcatheter aortic valve replacement in otherwise ineligible patients" (PMC4740457)* and standard practices, the transcaval access technique involves the following steps:

1. Pre-Procedural Planning

Imaging:

Perform contrast-enhanced CT angiography of the abdomen/pelvis to:

Identify the optimal puncture site between the IVC and aorta (typically L3–L4 vertebral level).

Measure the distance between the IVC and aorta (ideally <10 mm to minimize fistula risk).

Assess calcification, thrombus, or anatomic anomalies.

Note: In emergencies, fluoroscopic landmarks (e.g., vertebral bodies, renal arteries) may substitute for CT by performing venogram and arteriogram

2. Access Site Preparation

Femoral Venous Access:

Obtain ultrasound-guided access to the femoral vein (typically right-sided)

Insert a 8Fr venous sheath.

Arterial Access:

Place a 5–6Fr arterial sheath in the contralateral femoral artery or radial for aortic snaring and hemodynamic monitoring.

3. Caval-Aortic Crossing

Electrified Wire Crossing:

Under fluoroscopic guidance, using grandmother mother daughter technique advance a 0.014-inch electrified guidewire through a coaxial catheter assembly (composed of a 0.014 microcatheter, 0.035- microcatheter and RDC guide catheter) from the inferior vena cava (IVC) into the abdominal aorta.

Apply radiofrequency energy (50 W) to perforate the IVC and aortic walls. If severe calcification you can use higher energy i.e. 100 W

Aortic Snare Capture:

Deploy a nitinol snare (e.g., Amplatz GooseNeck®) sized to match the aortic diameter (1:1 ratio)

via the arterial sheath either femoral or radial to capture the wire in the aorta, creating a "body floss" through-and-through access.

4. Sheath Insertion

Exchange the electrified wire for a stiff guidewire (e.g.,Lunderquist wire).

Large-Bore Sheath Placement:

Advance a 22Fr sheath (e.g., Gore DrySeal®) over the wire into the aorta above the renal arteries. For Impella CP you need 16Fr sheath

5. Device Delivery

Deliver the intended device (e.g., Impella 5.0) through the sheath into the target location (left ventricle).

6. Closure

Nitinol Occluder Deployment:

Remove the sheath and deploy a nitinol vascular plug (e.g., Amplatzer™ Duct Occluder I) to seal the caval-aortic fistula.

Size the occluder to exceed the tract diameter by 2–4 mm usually 10 by 8 mm ADO 1.

Use an Ovation Abdominal Stent Graft or similar depending on aortic diameters for persistent bleeding or persistent shunting to avoid overloading the RV after deploying the nitinol vascular plug

Final Angiography:

Confirm closure with aortic and IVC angiography to rule out residual leaks.

7. Post-Procedural Care

Monitoring:

Observe for bleeding, hypotension, or hematuria (indicative of retroperitoneal hemorrhage).

Anticoagulation:

Administer aspirin for 6 months to prevent thrombus formation on the occluder.

Key Considerations

Expertise Required: Transcaval access demands proficiency in complex endovascular techniques. Proctoring is recommended for initial cases.

Risks: Aorto-caval fistula (5–10%), retroperitoneal bleeding (1–3%), and device migration.

Advantages: Avoids femoral/axillary artery complications, enabling MCS in patients with severe PAD.
